# Updating the Debate on Behavioral Competency Development: State of the Art and Future Challenges

**DOI:** 10.3389/fpsyg.2020.01267

**Published:** 2020-06-09

**Authors:** Sara Bonesso, Fabrizio Gerli, Rita Zampieri, Richard E. Boyatzis

**Affiliations:** ^1^Department of Management, Ca’ Foscari University of Venice, Venice, Italy; ^2^Department of Organizational Behavior, Weatherhead School of Management, Case Western Reserve University, Cleveland, OH, United States

**Keywords:** emotional, social and cognitive intelligence competencies, behavioral competencies, development, education, experiential learning

## Abstract

Emotional, social, and cognitive intelligence competencies have been recognized as the most critical capabilities for organizations to acquire at all levels. For this reason, a wide body of research since the 1980s has demonstrated their positive impact on individual performance, career success, and wellbeing across sectors and professional roles, and a large number of theoretical contributions on how these competencies can be effectively developed has emerged over time. We focus attention on the developmental and learning processes of emotional, social, and cognitive intelligence competencies that occur after formal training or educational courses provided by universities or certified organizations and directed to students or practitioners. Specifically, we conduct an exploratory literature review on the existing academic studies in order to identify the scholars and the pieces of research contributing most to the debate under investigation. This article aims at analyzing this body of research through a systematic review of the literature in order to: (i) provide a comprehensive critical analysis of the distinctive features of the theoretical and methodological frameworks adopted to develop these competencies; (ii) review the contexts in which the training initiatives analyzed by the literature have been delivered and the categories of learner involved; (iii) discuss the learning outcomes of these educational programs and how they have been assessed; (iv) identify gaps and inconsistencies in the current state of the literature, suggesting promising paths for future research; and (v) stimulate insights for educators, human resource managers, executives, and policymakers by organizing and critically analyzing the extant contributions on competency development. This review represents the first attempt to systematize the methodologies of the educational programs for competency development and to assess their effectiveness in order to assist educators and executives in their ongoing efforts to equip students and employees with the relevant skills needed to achieve superior performance in the workplace. At the institutional level, policymakers should promote a dedicated agenda with concrete actions to equip people with emotional and social intelligence competencies.

## Introduction

From recent employers’ surveys, behavioral or emotional intelligence competencies, also defined as ‘soft skills’ in the labor market’s jargon (Ritter et al., 2018), are becoming the most critical capabilities for organizations to acquire at all levels (LinkedIn, 2019; QS Intelligence Unit, 2019). These competencies are assuming a growing relevance for the workforce of the future, especially in the age of automation and artificial intelligence which are progressively replacing routine tasks and jobs (McKinsey Global Institute, 2018; Capgemini, 2019; World Economic Forum, 2019).

The literature that has extensively contributed to the debate on emotional, social, and cognitive intelligence has primarily identified three main theoretical streams and related measurement models (Ashkanasy and Daus, 2005; Cherniss, 2010; Walter et al., 2011; Boyatzis, 2018). The first stream conceives emotional intelligence as a mental ability which encompasses four branches, namely the ability to perceive emotions, the ability to use emotions to facilitate thought, the ability to understand emotions, and the ability to manage emotions (Mayer et al., 2000). The second stream considers emotional intelligence as a personality trait and the major contributions have been provided by the Bar-On’s (1997) model which contemplates five main components (intrapersonal skills, interpersonal skills, adaptability, stress management, and general mood) (Bar-On, 1997,2006) and by the Petrides and Furnham’s (2003) model which consists of four components (well-being, sociability, self-control, and emotionality). The third stream focuses on competencies as behavioral manifestations of emotional intelligence (Goleman, 1998; Boyatzis, 2018). Specific behavioral competencies are grouped into five main clusters: (1) the self-awareness cluster, which concerns knowing one’s internal states, preferences, resources, and intuitions, consisting of emotional self-awareness; (2) the self-management cluster, which enables the management of one’s internal states, impulses, and resources; (3) the social awareness cluster, which refers to how people are aware of others’ feelings, needs, and concerns; (4) the relationship management cluster, which includes abilities that induce desirable responses in others; and (5) the cognitive cluster, namely the ability to analyze information and situations.

Notwithstanding the ongoing debate on the different definitions and measurement models of emotional intelligence - which does not represent the topic of this article - empirical research has provided extensive evidence that the behavioral manifestation of emotional intelligence plays a crucial role in preparing individuals for contemporary careers, and subsequently in favoring their employability and entry-level success in the job market (Fugate et al., 2004; Hoover et al., 2010; Cortellazzo et al., 2020). This confirms that acquiring a proper set of emotional, social, and cognitive competencies during the education experience or in the preliminary stages of the career path can greatly enhance the chances of people’s success during the recruiting and selection process. Moreover, the aforementioned competencies are also labeled life skills (Gomes and Marques, 2013; Cronin et al., 2019), since they are required to deal with the demands and challenges of everyday life, and thus necessary along one’s career path to maintain employability over time and to attain career success (Amdurer et al., 2014).

However, academic studies and employers’ surveys have still revealed, on the one hand, a mismatch between the expectations of companies and the behavioral competency profile of candidates (Azevedo et al., 2012; QS Intelligence Unit, 2019), and on the other hand, a misalignment between the types of competencies learned by students attending higher education programs and those required by the labor market (Rubin and Dierdorff, 2009). This skills gap poses a challenge to organizations and to educational institutions, whose responsibility is to formally support the development of emotional and social competencies (ESCs) along with professional/technical skills. When in their book “Competence at Work,” Spencer and Spencer (1993: 12) stated that “you can teach a turkey to climb a tree, but it is easier to hire a squirrel,” they meant that behavioral competencies are more complex and difficult to develop than professional or hard skills; thus pursuing their development can imply challenges, such as the different role instructors have to assume, the active engagement of the learners, and the more experiential pedagogical approach teachers need to adopt (Bedwell et al., 2014).

Since behavioral competencies are currently the most in-demand skills in the labor market, and a wide body of research since the 1980s has demonstrated their positive impact on individual performance, career success, and wellbeing across sectors and professional roles (Boyatzis, 1982; Spencer and Spencer, 1993; Hopkins and Bilimoria, 2008; Koman and Wolff, 2008; Walter et al., 2011), a large number of theoretical studies on how these ESCs can be effectively developed has emerged over time. The purpose of this paper is to deliver a systematic review of the literature on this topic in order to make the following contributions: (i) provide the first comprehensive critical analysis of the distinctive features of the theoretical and methodological frameworks adopted for designing ESCs learning experiences; (ii) review the contexts in which these training initiatives have been delivered and the categories of learners involved; (iii) discuss the learning outcomes of the educational programs and how they have been assessed; (iv) based on the identification of knowledge gaps and inconsistencies in the current state of the literature, suggest promising paths for future research; and (v) stimulate valuable insights for educators, human resource managers, executives, and policymakers, by organizing and critically analyzing the extant contributions on competency development.

The paper is organized as follows. In the next section, the methods and the data set are introduced. Specifically, we describe the criteria adopted for selecting the articles that contributed to the debate on competency development. Subsequently, we describe the characteristics of the participants who attended the educational programs on ESCs. Furthermore, we analyze the different pedagogical frameworks and the methodological approaches implemented in training behavioral competencies, as well as the ESCs most frequently targeted by these programs. Then, we critically examine how the effectiveness of these educational initiatives has been measured. We conclude by discussing the gaps and the inconsistencies in the current literature, suggesting how future research should overcome them.

## Overview of the Method

The data collection process was preceded by a thorough preparatory stage, during which the authors conducted an exploration of the extant literature (Tranfield et al., 2003). This activity allowed the identification of the subject areas in which the debate has been developed and the keywords to use for the searching query. We decided to focus our attention on the developmental and learning processes of emotional, social, and cognitive competencies that occur after formal training or educational courses provided by universities or certified organizations and directed to students or practitioners. Our intention, in fact, is to disentangle the development of behavioral competencies as a result of on the job learning (Dreyfus, 2008) from the effects that training programs have on the same skills, by focusing on the latter.

We conducted an exploratory analysis using the following search query: [(“develop^∗^” OR “learn^∗^”) AND (“emotional intelligenc^∗^” OR “social intelligenc^∗^” OR “cognitive intelligenc^∗^” OR “social competenc^∗^” OR “emotional competenc^∗^” OR “cognitive competenc^∗^” OR “behavioral competenc^∗^” OR “behavioral competenc^∗^” OR “behavioral skill^∗^” OR “behavioral skill^∗^” OR “emotional capabilit^∗^” OR “social capabilit^∗^” OR “cognitive capabilit^∗^” OR “behavioral capabilit^∗^” OR “behavioral capabilit^∗^” OR “emotional skill^∗^” OR “social skill^∗^” OR “cognitive skill^∗^”) AND (“education”)]. We searched the terms in the keywords, titles, and abstracts of documents contained in two databases, Scopus and Web of Science, recognized by the scientific community as having wide coverage (Zupic and Èater, 2015). Moreover, we limited our research to articles published in English and to the three subject areas that were shown to welcome the largest part of the debate, namely business and management, psychology, and social sciences. The business and management field was included to capture development programs aimed at students and practitioners which have an impact on organizations through their actual or future human capital. The psychology area was considered with the aim of acquiring a deep understanding of the psychological dimensions, processes, and outcomes that characterize behavioral competencies training and development programs. Finally, we considered the social sciences area in general in order to include those contributions neglected by the other categories, such as studies in learning and education.

Since the aim of our review is to contribute to the existing academic literature while relying on well-established and impactful knowledge, we decided to limit the analysis to peer-reviewed academic articles. Indeed, the search query was defined to exclude conference papers, books, and book chapters. Applying the aforementioned keywords, Scopus and the Web of Science databases retrieved 6,106 unique articles, after checking for duplications.

Starting from the consideration that medium to top journals publish works that have an impact on academic research, we refined our review by considering only studies published within the first 300 journals in the SCImago list in the areas of business and management, psychology, and social sciences. The titles and abstracts of these articles were checked to assess their fit with our research purposes.

In so doing, we also applied some exclusion criteria and boundary conditions. Firstly, we excluded from the data set those articles focused on children and elderly people as target subjects, since our interest is oriented toward research dealing with the development of ESC competencies in people of working age or approaching the labor market. As a result, we focused on studies centered on adolescents/students and adults. We also excluded studies dealing with physical and behavioral disorders, such as autism, anxiety, and depression. The reason lies in the consideration that a focus on these pathologies can alter the design of the program, whose aim is to cope with these disturbances, and consequently the effectiveness of the learning outcomes. Finally, we excluded research conducted at the group or organization level of analysis, while considering those studies centered on individuals’ competency development. We did not insert any time limit.

This selection process allowed us to collect a final sample of 71 articles published within the period 1982–2020. This selection process further allowed the identification of the scholars who contributed the most to the debate under investigation.

**FIGURE 1:**
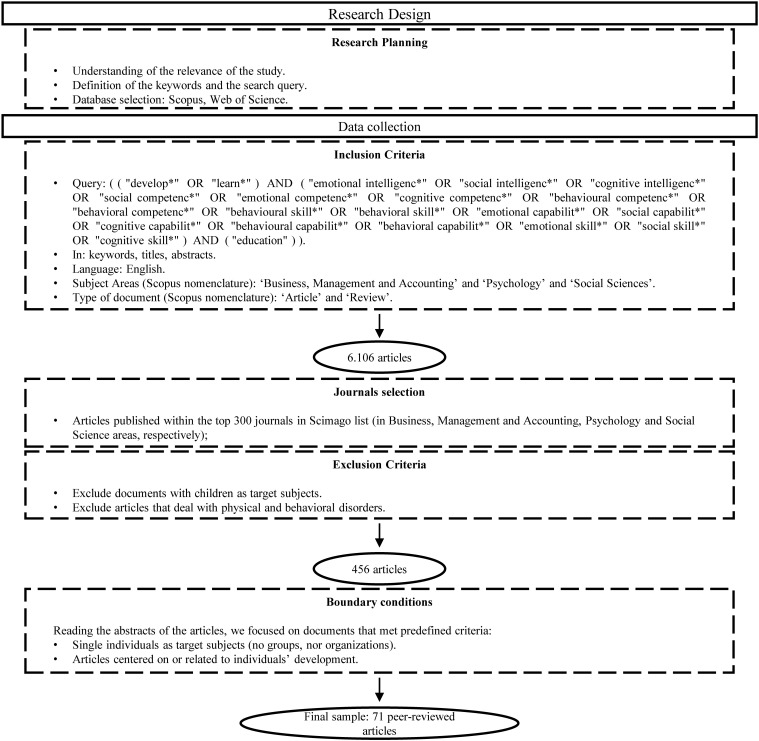
Research design.

Figure 1 provides a visual representation of the data collection flow.

## Data Set Description and Data Analysis

The final sample consists of 71 peer-reviewed articles, 38 of which present or test the effectiveness of programs that develop behavioral competencies. The remaining works encompass both theoretical and empirical studies aimed at (i) reviewing different learning approaches for enhancing ESCs (e.g., Hoffman, 2009; Meiklejohn et al., 2012; Ott and Michailova, 2018); (ii) testing the impact of competency development on variables such as life satisfaction, stress reduction, performance (see, for instance, Camuffo et al., 2009; Amdurer et al., 2014; Dave et al., 2019); and (iii) valuing or discussing the impact and use of different tools and methods for the aforementioned purposes (e.g., Leonard, 2008; Wheeler, 2008; Boyan and Sherry, 2011; Miller et al., 2012).

For each of the 71 articles, we examined the theoretical frameworks adopted, coherently with the different streams of research illustrated in the introduction section. From this analysis emerged that more than half of the works (54%) explicitly referred to the behavioral approach to emotional and social intelligence competencies, whereas, respectively, 7% and 6% of the articles pertain to the mental ability and the trait models. A few articles combine the different streams of research (2%), or adopt other theoretical framework such as the cultural intelligence approach (4%) or the cognitive skills (6%). The remaining 21% of the articles do not explicitly refer to a theoretical model, focusing instead on the impact of the training programs on few specific skills measured through disparate self-rating scales. This evidence shows the emphasis ascribed to the behavioral stream and to the other self-report approaches in addressing the issue of emotional and social intelligence development. As previously discussed in the literature (Walter et al., 2011), this can be due to the challenges posit by the other approaches and associated measures used for the assessment of emotional and social intelligence.

Focusing on the contributions illustrating competency development programs, extant research suggests that developing behavioral competencies requires pedagogical approaches and related didactic techniques which actively engage the learner (Hoover et al., 2010). People, and adults in particular, learn what they want to learn and, as a consequence, individuals who want to develop their ESC competencies should be conscious of what, how, and for what purposes they want to change or improve their set of behaviors (Boyatzis, 2008). We treated learners’ awareness as a discriminant factor when we analyzed the contributions presented by the educational programs.

The results of the analysis suggest that literature splits into two broad categories. The articles included in the first category describe and test the effectiveness of courses and programs designed and developed with the clear purpose of improving behavioral competencies. This is the case of the Leadership Assessment and Development (LEAD) course implemented in an MBA curriculum at Case Western Reserve University (Boyatzis and Saatcioglu, 2008; Rhee, 2008; Passarelli et al., 2018) or the Emotional Intelligence Training Program (EITP) (Gilar-Corbí et al., 2018), to cite just two. Table 1 summarizes these works and their main findings.

**TABLE 1:** Courses designed for ESCs development.

Study	Main findings
Ballou et al. (1999)	The Professional Fellows Program for midcareer professionals is a learner-centered, non-graded program that provides participants with the tools for understanding and facing current leadership challenges, while encouraging the creation of a personalized learning agenda and a network of peers that promote lifelong learning. An assessment of the program is provided along with its description.
Barbera et al. (2015)	The application of a whole person learning approach to family business education proves its effectiveness for developing the ESC competencies of the next generation of entrepreneurs.
Boyatzis et al. (2002)	The article combines cross-sectional and longitudinal data on multiple cohorts of MBA attendees. Results show that emotional and social competencies can be improved in MBA programs that are not only based on traditional knowledge acquisition methods, but that adopt a more holistic approach to students’ development.
Bücker and Korzilius (2015)	The Ecotonos cross-cultural simulation game helps increase the metacognitive, motivational, and behavioral components of participants’ cultural intelligence.
Camuffo et al. (2009)	The article examines the learning that occurred during an MBA, measured in terms of competency development and its impact on career advancement and compensation.
Dowling et al. (2019)	The MindOut social and emotional learning program targeting adolescents shows its effectiveness for improving students’ emotional and social skills and wellbeing.
Filella et al. (2018)	The Happy emotional education program is a gamified tool that supports adolescents’ emotional competency development.
Gilar-Corbí et al. (2018)	The Emotional Intelligence Training Program, in its online, in-class, and coaching versions, can effectively improve students’ emotional intelligence competencies.
Griffith (1999)	The reflecting team case model is presented and described as an alternative didactic method for improving participants’ ESC intelligence competencies.
Harris et al. (2016)	The Community Approach to Learning Mindfully (CALM) program for educators proves its effectiveness in promoting teachers’ mindfulness and emotional functioning, with effects in terms of individuals’ wellbeing.
Havighurst et al. (2015)	The Tuning into Teens (TINT) parents’ emotion socialization program reveals an effective course for improving parents’ emotional responses to their adolescent children’s behaviors, while reducing family conflicts.
Hoover et al. (2010)	The study presents, discusses, and assesses the effectiveness of the whole person learning approach, confirming its soundness for developing individuals’ ESC intelligence competencies.
Jennings et al. (2019)	The Cultivating Awareness and Resilience in Education (CARE) mindfulness-based program for teachers enhances teachers’ mindfulness and emotion regulation, and reduces the level of their distress.
Leventhal et al. (2015)	The Girls First Resilience Curriculum that targets girls in high poverty conditions displays its effectiveness for improving participants’ emotional resilience, social and emotional assets, self-efficacy, and psychological and social wellbeing.
Mirvis (2008)	The paper describes the features and use of consciousness-enhancing experiences for executives.
Passarelli et al. (2018)	A longitudinal assessment of the Leadership Assessment and Development (LEAD) course implemented in an MBA program has been used by the authors to understand how patterns of competency development change over time and what factors intervene in this change. The authors identify five factors that affect competency development.
Ramsey and Lorenz (2016)	Cross-cultural management education increases students’ cultural intelligence, along with their commitment and satisfaction with the course.
Rhee (2008)	This longitudinal study is based on the Leadership Assessment and Development (LEAD) course implemented in an MBA program for understanding the dynamics of ESC competency development. Results indicate that different ESC competencies are developed at different periods throughout the course.
Rhee and Sigler (2010)	The article presents the features of the MS program in Executive Leadership and Organization Change (ELOC), which is based on the action learning, competency development, and teamwork pedagogical framework. The program aims to develop leaders who provide outstanding performance.
Schoeps et al. (2019)	The PREDEMA 2.0 program targeting university students improves students’ emotional competencies and wellbeing in the short run.
Schoeps et al. (2018)	The PREDEMA program aims to boost adolescents’ emotional competencies in order to promote their wellbeing and improve the quality of relationships they build with their peers. The program is effective for reducing cyberbullying behaviors.
Sheehan et al. (2009)	Adopting a classroom-as-organization (CAO) approach significantly contributes to the development of students’ emotional intelligence competencies.
Stoller (2013)	The Cleveland Clinic Academy leadership development course is used as a point of reference in providing guidelines for leadership development in healthcare.
Trautwein et al. (2020)	The authors test and confirm that different types of mental training intervention (targeting attention, socio-affective skills, and socio-cognitive competencies) intervene in different areas of the human brain, thus affecting people’s behaviors.
Wallin et al. (2007)	Simulations are effective methods for developing teamworking and team coordination capabilities in medical emergency teams.

In the second category, we considered those programs for which the improvement of individuals’ behavioral skills is a collateral, although welcome, byproduct of courses designed with different learning objectives. A synthesis of these studies is provided in Table 2. For instance, in a recent longitudinal study, Gong et al. (2016) show that attending pre-school has long-term effects on the development of Chinese adolescents’ social skills. Similar findings are discussed by Oi (2019), who discovered that adolescents advancing in formal education improve their non-cognitive skills, an umbrella term that indicates competencies such as conscientiousness, self-efficacy, and future orientation. More generally, and moving to graduate students, it has been found that MBA programs support the development of attendees’ emotional intelligence (Thompson et al., 2019).

**TABLE 2:** Studies designed with different purposes that develop ESCs as a byproduct.

Study	Main findings
Arco-Tirado et al. (2011)	The peer-tutoring program (PTP) positively influences tutors’ social skills.
Bonesso et al. (2015)	Academic courses relying on traditional learning methods should complement these methods with individual experiential learning and social experiential learning to develop students’ ESC skills.
Burch et al. (2016)	In courses aimed at enhancing declarative and procedural knowledge, students’ attending face-to-face, instead of web-based, classes report higher levels of cognitive and social competencies.
Farashahi and Tajeddin (2018)	Students assess simulation as the most effective method for developing interpersonal skills and self-awareness. Simulation and case study are believed to improve students’ problem-solving competencies more than lectures.
Gong et al. (2016)	Pre-school attendance has a long-term impact on adolescents’ social skills.
Johannisson (1991)	Academic entrepreneurial programs should not only focus on teaching the ‘know what’ (content knowledge), but also work to develop the ‘know why’ (attitude and motivational skills) and the ‘know how’ (social skills).
Mendo-Lázaro et al. (2018)	A cooperative team learning approach positively affects the development of students’ social competencies.
Morris et al. (2005)	The incorporation of elements of the liberal arts, especially visual arts and poetry, can enhance students’ self-awareness and recognition of their emotions.
Oi (2019)	Continuing formal education after adolescence and young adulthood improves persons’ non-cognitive skills such as conscientiousness and future orientation.
Riggs et al. (2010)	Youths participation in after-school programs positively impacts on their concentration and emotion regulation skills.
Ruiz-Ariza et al. (2018)	Adolescents who participate in an 8-week training using Pokémon-Go display improvement in selective attention, concentration, and sociability levels.
Thompson et al. (2019)	MBA programs support participants in developing their emotional intelligence competencies.
Turner and Mulholland (2017)	Enterprise education equips students with behavioral competencies such as self-confidence, communication capabilities, creative thinking, and problem solving.
Zoller (1999)	Teaching strategies relying on inquiry-oriented class discussions, *ad hoc* examination practices, and evaluations by both peers and graduate students are effective in developing students’ higher-order cognitive skills (HOCS) (i.e., decision-making, problem solving, critical thinking).

The following sections will present the results of the analyses we conducted on the selected articles. We will focus our attention on the characteristics of participants in educational programs, describe the methodological approaches that constitute the theoretical foundations of each program, evaluate the nature of the developed competencies, and conclude with remarks on the effectiveness of the considered programs in light of their purposes.

### Participants Involved in Competency Development Programs

Our analysis of the articles in our data set, considering both programs specifically designed to develop ESC competencies and those designed with different purposes, allows us to identify three groups of participants considered by the extant research on behavioral competency development: (i) youths and adolescents, mostly high school students (the sampled individuals in this category are 12 to 18 years old); (ii) undergraduate and graduate students, also encompassing full-time MBA attendees; and (iii) adults and professionals, namely people who are actively employed in a working position and for which universities and/or organizations offer training programs.

Research targeting youths and adolescents is focused on two fundamental aspects: individuals’ well-being (see, for instance, Leventhal et al., 2015; Dowling et al., 2019) and promotion of the coexistence of youths with their peers (e.g., Schoeps et al., 2018). The development of behavioral competencies is believed to support adolescents in coping with the transitions that occur during this age and that are characterized by many possible stressors (Dowling et al., 2019). The process of maturation in fact brings both biological and psychosocial changes that affect the lives of youths and adolescents. It is during this period that individuals start to construct their identity, experiencing conflicts with their family, seeking acceptance among their peers, and laying the foundations of their future lives and careers (Dowling et al., 2019). “Programs aim[ing] to enhance adolescents’ social and emotional skills […] act as protective factors for promoting positive wellbeing and reducing the onset of mental health difficulties and health risk behaviors” (Dowling et al., 2019: 1258). Furthermore, encouraging the development of adolescents’ behavioral competencies is a strategic action that can improve the co-existence of youths with their peers and prevent the occurrence of bullying behaviors (Filella et al., 2018; Ruiz-Ariza et al., 2018; Schoeps et al., 2018). Emotional and social competency development programs are meant to provide individuals with the tools to better interact and relate with their peers and teachers, while improving the recognition and management of their emotions to reduce conflicts and aggressiveness (Schoeps et al., 2018).

The second identified category considered students who attend competency development programs in the higher education setting. The motivations behind the design of the development programs presented by these works lead us to distinguish between two subgroups of target subjects: undergraduate/graduate students and MBA attendees. As suggested by Schoeps et al. (2019), individuals leaving the high school world and entering a university career can go through a period of stress due to the increased amount of responsibilities and expectations coming from the environment. They become independent, moving from their hometown or home country; they are required to build social relationships and to face the academic challenges while coping with the competition of the university environment. They also bear the burden of imagining and projecting their lives after graduation.

Emotional training programs aimed at developing students’ behavioral competencies can support individuals going through these unavoidable steps, with a positive impact on their wellbeing (Schoeps et al., 2019). In addition, university programs have to prepare students for the job market. The current business landscape asks new job market entrants to be effective in translating the concepts and behaviors learned during the academic path into an organizational reality characterized by complexity and dynamicity (Gilar-Corbí et al., 2018). Furthermore, globalization asks workers to interact and collaborate with people from different countries and cultures (Bücker and Korzilius, 2015). It is not surprising that the development of cultural intelligence has entered educators’ learning agendas during the last decades, and it has become the focus of a number of educational programs (Ramsey and Lorenz, 2016). Cultural intelligence is defined as “a person’s capability for successful adaptation to new cultural settings, that is, for unfamiliar settings attributable to cultural context” (Earley and Ang, 2003: 9).

Furthermore, organizations seek people with sound social skills who are able to enrich and enhance their own and the organization’s knowledge and capabilities through lifelong learning (Mendo-Lázaro et al., 2018). In this scenario, the development of emotional and social competencies becomes of paramount importance (Bücker and Korzilius, 2015; Gilar-Corbí et al., 2018; Mendo-Lázaro et al., 2018). This explains the presence of competency development courses within MBA programs whose aim is to prepare the future managers and leaders (Boyatzis et al., 2002; Passarelli et al., 2018; Thompson et al., 2019), who should impact and transform their organizations and communities for the better (Rhee and Sigler, 2010).

It is well established in the academic literature that advanced professionals, and adult learners in general, have different learning needs compared with young people or university students (Ballou et al., 1999). The articles that belong to the third category highlight the role that ESC competencies play in particular groups of subjects: educators and managers.

Parents, as educators, play a key role in influencing their children’s emotional development (Havighurst et al., 2015). For this reason, it is of paramount importance that parents learn to recognize and manage not only their own and but also their children’s emotions. In youths’ development, another key role is played by teachers. They are responsible not only for the quality of education, but also for providing stimuli and learning opportunities that boost students’ curiosity and performance (Harris et al., 2016; Jennings et al., 2019). However, according to Jennings et al. (2019), about 50% of K-12 teachers in the US attest to being under pressure and experiencing a lot of stress in relation to their job. The causes are attributable to factors such as being constantly under the judgment of students, parents, and school principals; being asked to manage students’ misbehaviors, emotional reactions, and scarce interest in school topics; and feeling unable to perform a job in which there is no working time and for which they need to take the work home (Meiklejohn et al., 2012; Harris et al., 2016; Jennings et al., 2019). Programs that aim to reduce teachers’ stress have been designed and implemented to support teachers in coping with these possible critical situations. Reducing teachers’ stress is a key step in the creation of a more relaxed and enjoyable climate in class, which has an impact on students’ emotional responses to in-class activities and on their performance (Harris et al., 2016; Jennings et al., 2019).

In the organizational setting, considerable efforts and resources have been spent on development programs that target managers’ competency portfolio. Universities and business organizations acknowledge that having great leaders contributes to organizational success and is instrumental in the achievement of a sustainable competitive advantage. In this scenario, the development of ESC competencies sustains managers’ outstanding performance and supports them in facing the challenges of the current business environment (Mirvis, 2008; Kark, 2011). Surprisingly, in the articles analyzed, we found that some studies have primarily focused only on educational programs delivered to professionals operating in the healthcare sector. In this particular setting, most managerial and leading positions are assigned in response to academic performance or cognitive ability. However, being effective doctors, medical researchers, or nurses does not necessarily reflect an effective manifestation of social and emotional competencies (Stoller, 2013). Successfully managing, coordinating, inspiring, and working in teams are crucial behaviors both for facing emergencies and coping with the everyday challenges that hospitals and clinics face. In such complex organizations, where there are a lot of stakeholders with different roles and requirements, leaders are asked to take forward a culture of effective coordination and to manage the trade-off between costs, access, and quality of health services (Wallin et al., 2007; Stoller, 2013). These skills are the products of learning and practicing efforts trained by *ad hoc* courses such as team coordination programs (Wallin et al., 2007).

### Pedagogical Frameworks

This section presents the results of the analysis we conducted on the theoretical foundations and methodological approaches of the didactic programs purposely designed to enhance behavioral competencies (Table 1). Thus, we did not consider those studies in which the competency development was an unintentional outcome of the learning experience (Table 2).

A common thread characterizes these development programs: despite relying on different frameworks, these studies agree on the importance of experiential learning methodologies to improve behavioral skills. The review shows that two main theoretical approaches are extensively adopted in designing these programs. The first is Kolb’s experiential learning theory (Kolb, 1984), furtherly advanced by programs like the Leadership Assessment and Development (LEAD) course (Boyatzis et al., 2002; Rhee, 2008; Passarelli et al., 2018), or the Executive Leadership and Organization Change (ELOC) program (Rhee and Sigler, 2010). According to Kolb, the process of learning from experience encompasses an understanding and re-elaboration of new experiences that adds to previous ones, modifying and enriching them. The experiential learning cycle explains this process and depicts the four sequential phases through which individuals elaborate and internalize experiences: (i) concrete experience of a new behavior, thought or action; (ii) reflective observation of how the individual or other people behaved or acted in the same situation or under similar conditions; (iii) abstract conceptualization of the outputs of these actions or behaviors, in terms of both individuals’ feelings and cause-effect relationships; and (iv) active experimentation of the experience enriched by a new conceptualization of the same (Kolb and Kolb, 2009).

The second approach is the whole person learning pedagogy (Rogers, 1980), which has been applied in programs illustrated, for instance, by Hoover et al. (2010) and Barbera et al. (2015). Specifically, Hoover et al. (2010) describe the approach they implemented in an MBA course to respond to the needs of enterprises that seek employees who possess teamworking, communication, conflict resolution, and problem-solving skills. When accompanied by an assessment of the ESC competencies, the whole person learning pedagogy is revealed to be an effective method for acquiring and retaining skills that allow executives to be successful in their roles. This approach is an extension of the concept of whole person learning presented by Rogers (1980). In particular, it is a learning pedagogy that impacts not only on people’s cognitive capabilities, typically involved in a learning process, but also on their emotions and behaviors: hence the term ‘whole person.’ Involvement of the cognitive, emotional, and behavioral dimensions supports engagement and motivation toward the learning process (Hoover et al., 2010). This pedagogy has proved to be effective both when implemented into MBA courses and for developing the skills of undergraduate students involved in their family businesses (Hoover et al., 2010; Barbera et al., 2015).

Further support for these results is provided by scholars who focus on programs that strongly rely on experiential methods for developing teachers’ competencies such as self-awareness, self-control, resilience, empathy, and compassion (Meiklejohn et al., 2012, Jennings et al., 2019). Finally, Bücker and Korzilius (2015); Ramsey and Lorenz (2016), and Ott and Michailova (2018) contend that experiential training is of paramount importance when developing managers and, more generally, practitioners’ cultural intelligence competencies.

Literature introducing and testing the effectiveness of programs for ESCs development draws on a number of different experiential and person-centered methodologies that constitute the application of different learning techniques. Based on the methodology implemented in the educational program, we group these studies as follows: programs based on the intentional change theory (ICT; Boyatzis et al., 2002; Rhee, 2008; Passarelli et al., 2018); on the social and emotional learning (SEL) approach (Cristóvão et al., 2017; Taylor et al., 2017; Filella et al., 2018; Schoeps et al., 2018, 2019; Dowling et al., 2019); on mindfulness-based programs (Meiklejohn et al., 2012; Davis, 2014; Harris et al., 2016; Jennings et al., 2019; Trautwein et al., 2020); on cultural intelligence development courses (Bücker and Korzilius, 2015; Ramsey and Lorenz, 2016; Ott and Michailova, 2018), and on other pedagogies which rely on experiential learning such as consciousness-raising experiences and the classroom-as-organization approach (Mirvis, 2008; Sheehan et al., 2009). The rest of this section is devoted to describing in more detail the differences among these methodological approaches and the behavioral competencies nurtured by these programs.

Originally defined as self-directed learning theory, the ICT describes the essential components that lead an individual toward a desirable and sustainable change in his behaviors, thoughts, feelings, and perceptions (Boyatzis, 2008). The LEAD program (Boyatzis et al., 2002; Boyatzis and Saatcioglu, 2008; Rhee, 2008; Passarelli et al., 2018), implemented in an MBA curriculum since 1987 at Weatherhead School of Management, Case Western Reserve University, follows the ICT pedagogy to support students in the development of their emotional and social competencies.

In harmony with Boyatzis’ ICT, the course is structured to guide participants throughout five phases, also defined “discoveries” or “epiphanies”: (1) identification of the ideal self and development of a personal vision; (2) discovery of the real self; (3) creation of a personal learning path; (4) the practice of desired behaviors, which includes reflections on actions and feelings; and (5) the creation of relationships that support individuals during the training phase, such as the involvement of participants in coaching sessions with trained professional coaches (Passarelli et al., 2018). Inspired by the LEAD course, the Professional Fellows Program is aimed at midcareer professionals who are entering managerial positions. The program guides participants onto a path where they are introduced to leadership notions and challenges, and they have also the opportunity to assess their competencies, develop their learning agenda, and construct a network with other students, with the aim of sustaining each other’s lifelong learning commitment (Ballou et al., 1999). Both LEAD and the Professional Fellows Program integrate experiential learning theory with the whole person learning approach (Ballou et al., 1999; Boyatzis and Saatcioglu, 2008).

Social and emotional learning (SEL) is a concept born in 1994, when a group of researchers and professionals met at the Fetzer Institute (Kalamazoo, MI, United States) to discuss the importance and opportunity of inserting the development of social and emotional competencies into school curricula. In the same year, Daniel Goleman and Eileen Growald, who participated in this meeting, founded the Collaborative for Academic, Social, and Emotional Learning (CASEL), whose aim is to support the inclusion of SEL for scholars and teachers (Cristóvão et al., 2017). SEL is defined as “a strategy to nurture students’ social and emotional competences by way of explicit teaching. SEL uses a student-centered approach that encourages student participation in the learning process and in the development of analytical communication and collaborative behaviors” (Cristóvão et al., 2017: 2).

The SEL approach contends that schools should explicitly teach emotional and social competencies, along with the cognitive ones already taught, applying a more holistic approach through which the learning of these skills occurs not only in class but also through the involvement of families and communities. In this scenario, a key role is played by teachers, who become the promotors and the cornerstone in the implementation of SEL’s purposes (Cristóvão et al., 2017). The SEL approach targets five ESC competencies: self-awareness, self-management, social awareness, relational competencies, and responsible decision-making, integrating the teaching of these skills into school curricula and practices (Cristóvão et al., 2017; Taylor et al., 2017). An example is provided by the MindOut program, developed to support older adolescents’ emotional and social development, and designed according to CASEL’s theoretical framework. This program is based on a number of experiential learning practices such as collaborative learning, creation of scenarios, games, and simulations, and it adopts a holistic approach to continuing the training outside the classroom and at home (Dowling et al., 2019).

Another SEL program that targets adolescents is presented and tested by Schoeps et al. (2018). This program, named PREDEMA, aims to enhance the understanding and management of students’ emotions, contributing to build a positive climate in the classroom. Indeed, PREDEMA’s ultimate goal is to improve students’ co-existence and to facilitate the relationships between students and teachers, while reducing cyberbullying behaviors and enhancing students’ wellbeing in the long run. The program is based on the four-branch model of emotional intelligence (Mayer et al., 2016) describing and categorizing the emotional intelligence skills into: (1) the ability to perceive emotions; (2) the ability to use emotions to sustain the cognitive process; (3) the ability to understand or ‘label’ emotions; and (4) the ability to manage emotions. The first group of skills (perceiving emotions) contains competencies that reflect a lower level of complexity. When moving to the other groups, however, the tasks become more challenging and the competencies more complex. The PREDEMA program, as well as its 2.0 version targeting university students (Schoeps et al., 2019), has its roots in the dialogical paradigm of learning, wherein the learning process is the result of communicative actions between learners and the subjects with which they interact (Racionero and Padrós, 2010). Moreover, responding to a need to boost emotional education, Filella et al. (2018) present and evaluate the effectiveness of Happy Software 12–16. This gamified program was developed to support adolescents in the management of their emotions. It is based on the model of emotional competencies presented by the Group of Psychopedagogical Orientation, which defines five categories of emotional skills: emotional awareness, emotion regulation, autonomy (e.g., self-esteem, motivation, resilience), social skills, and life competencies (e.g., adaptivity, seeking help, personal wellbeing, ability to contribute to community, society’s wellbeing) (Filella et al., 2018).

Mindfulness-based interventions (MBIs) represent another set of practices adopted to nurture behavioral competencies. Mindfulness is defined as the awareness that derives from purposefully and non-judgmentally paying attention to current experiences while maintaining “openness, curiosity, and acceptance toward [these] experiences” (Meiklejohn et al., 2012; Harris et al., 2016: 144). It has found a plethora of applications and related exercises, such as breathing meditation, body scan, loving kindness meditation, and yoga, among others, to cultivate in participants present moment attention and self-awareness, but also affective qualities of care, gratitude, and loving kindness, as well as social awareness through perspective taking (Meiklejohn et al., 2012; Davis, 2014).

The strength of mindfulness-based programs is that they create the mental conditions under which behavioral competencies can be manifested and enhanced. These programs support trainees’ resilience and wellbeing, reduce stress, improve the way people relate and interact with others, and promote people’s commitment to change (Meiklejohn et al., 2012; Davis, 2014). Mindfulness-based programs intervene in the brain’s plasticity—specifically, in the areas of the brain devoted to emotion regulation (Meiklejohn et al., 2012)—and contribute to enhancing competencies such as empathy, creative thinking, teamworking and collaboration, social awareness, and self-awareness, to name a few (Meiklejohn et al., 2012; Davis, 2014; Jennings et al., 2019). Besides the well-known mindfulness-based stress reduction program (MBSR) (Kabat-Zinn, 1990), a number of programs are mentioned, tested, and analyzed in the literature we reviewed. Harris et al. (2016), for instance, test the efficacy of a yoga-based intervention, the Community Approach to Learning Mindfully (CALM), to support educators’ wellbeing. According to the authors, mindfulness, along with other contemplative practices, is at the base of yoga exercises.

In a literature review published in 2012, Meiklejohn and other scholars compare the effectiveness and features of three mindfulness-based experiential training programs aimed at improving teachers’ resilience and wellbeing: the Mindfulness-Based Wellness Education (MBWE) program; Stress Management and Relaxation Techniques (SMART) in education; and the Cultivating Awareness and Resilience in Education (CARE) program. In this last case, Jennings et al. (2019) empirically validate the positive effects of the program on teachers’ mindfulness, distress, and emotion regulation. Finally, a recent study by Trautwein et al. (2020) has tested and confirmed that different types of contemplative or meditation-based practices induce plasticity in different areas of the human brain, suggesting to researchers and practitioners the need for more exploration of targeted interventions devoted to developing different types of skill.

A group of articles included in our analysis focus on the development of cultural intelligence competencies, confirming that, as happens for emotional intelligence skills, cross-cultural cognitive and behavioral capabilities can be improved through dedicated training and development programs (Ramsey and Lorenz, 2016). Scholars contend that courses that embrace experiential learning principles show their effectiveness in developing cultural intelligence, especially when combined with experiences abroad and interactions with people from different countries (Ott and Michailova, 2018). This is confirmed by Bücker and Korzilius (2015), who tested the effectiveness of a cross-cultural simulation-based game, Ecotonos. Moving from contributions that support the need to complement knowledge acquisition with experiential learning practices, this study supports the effectiveness of games as a dimension for training new cultural behaviors in a safe environment. Games, in fact, allow evaluation of the consequences of practiced behaviors and the implementation of corrections if such behaviors do not provide the expected results (Bücker and Korzilius, 2015).

Consciousness-raising experiences are practices that have their roots “in sociopolitical discourse and empowerment movements (Freire, 1972), feminist theory and study groups (Shreve, 1990), myriad forms of psychotherapy (Prochaska, 1979), and many spiritual disciplines (Irwin, 2002)” (Mirvis, 2008: 174). However, they prove their effectiveness even when applied to the business context (Mirvis, 2008). Consciousness-raising experiences in organizational environments refer to a set of activities, usually part of leadership development programs, that aim at cultivating executives’ self-and social awareness. They can be in the form of sessions/meetings in which people speak about their lives, work, aspirations, what motivates and guides them, and the challenges they face. They can also translate into shared experiences, such as outdoor activities or team challenges that promote participants’ self-reflection, stimulating their cognitive and emotional intelligence competencies.

An example of a consciousness-raising experience is service learning (Mirvis, 2008). In service learning programs, students or course participants take part in community service for learning and development purposes. Answering the question of what it takes “to develop enlightened leaders who can transform their organizations and communities,” Rhee and Sigler (2010: 163) describe a leadership development program based on service-learning: the ELOC course. The ELOC program, like other service-learning based courses, naturally integrates emotional and social competencies development to sustain leaders in their effectiveness. It is based on the action learning, competency development, teamwork (ACT) philosophy. The program is designed to actively involve participants and guide them in putting their competencies into practice. At the same time, they are stimulated to reflect on their actions and make corrections when necessary, in view of continuous personal improvement. This first stage is inspired by Kolb’s experiential learning cycle (Kolb and Kolb, 2005) and in line with the double-loop learning theory (Argyris and Schon, 1978; Argyris, 1991). As for the competency development, the ELOC program provides students with the tools to assess their emotional and social competencies while they are guided to develop a customized and personalized learning plan. Moreover, students are divided into groups according to their learning styles (through the Kolb Learning Style Inventory), and they are challenged to practice their teamworking abilities and provide feedback in order to contribute to each other’s development (Rhee and Sigler, 2010).

A similar approach is employed in the health care leadership development program offered by the Cleveland Clinic Academy. The course articulates the development of leadership competencies through three complementary experiences: (i) lectures, in which participants learn the characteristics of an effective and outstanding leader; (ii) mentoring and coaching sessions, in which they receive and ask for feedback; and (iii) opportunities to practice the competencies that participants want to develop (Stoller, 2013).

Another experiential learning methodology is the classroom-as-organization approach (CAO), wherein Sheehan et al. (2009) tested the positive effects on improving behavioral competencies on a sport management event course. The CAO approach is an experiential learning pedagogy that stimulates students through activities such as simulations, role play, teamwork, and group case studies. Mimicking a real working environment, participants are exposed to the acquisition of conceptual knowledge on the topic of their academic course and are provided with the opportunity to develop their behavioral skills (classified into self-awareness, self-management, social awareness, and relationship management).

Sheehan et al. (2009) first explore the connection between the CAO approach and the development of ESC competencies, treating the CAO methodology as an opportunity to test whether students’ emotional intelligence competencies can be improved “without formally instructing them on emotional intelligence theory in the classroom” (Sheehan et al., 2009: 79). Students are also stimulated in reflective practices (keeping a personal journal, through meetings, and writing a final essay on their experience) in order to evaluate their personal experiences.

Scholars cite cooperative learning as an effective methodology for collectively learning new concepts. In a recent work, Mendo-Lázaro et al. (2018) contend that when cooperating, students can also practice their social skills. For this reason, the authors suggest that a cooperative learning methodology can constitute an effective training stage for the development of social competencies in students once the trainees have been informed of the characteristics of ‘proper’ social behaviors during lectures and seminars (Mendo-Lázaro et al., 2018). A similar approach is described for the training of trauma teams in hospitals. The skill of teamwork, necessary for managing emergencies, is developed through experiential learning methods, in particular constructing scenarios. First, participants attend lectures to learn the characteristics of effective technical and teamworking behaviors; they are then challenged to put these behaviors into practice during the aforementioned simulations. Finally, feedback is provided during the simulations and at the end of the training experience (Wallin et al., 2007).

### Behavioral Competencies as Learning Objectives

The aforementioned programs have been also analyzed in consideration of the behavioral competencies that they aimed at enhancing, regardless of their effectiveness in terms of learning outcomes. For each educational program, Table 3 reports a short description of the learning objectives stated in the study.

**TABLE 3:** Programs’ learning outcomes.

Educational Program	Study	ESCs
CALM	Harris et al. (2016)	Positive emotions (compassion, acceptance); self-awareness; self-control and emotion regulation; sense of community
CARE for Teachers	Jennings et al. (2019)	Emotional support; self-awareness; self-control; and emotion regulation
Classroom-as-organization approach	Sheehan et al. (2009)	Self-awareness competencies, self-management competencies, social awareness competencies and relationship management competencies
Consciousness-raising experiences	Mirvis (2008)	Self-awareness; social awareness; dealing with diverse people; promoting inclusiveness; relating to society
Cross-cultural management course	Ramsey and Lorenz (2016)	Cultural intelligence (cognitive, metacognitive, motivational, and behavioral components)
Ecotonos cultural simulation game	Bücker and Korzilius (2015)	Cultural intelligence (cognitive, metacognitive, motivational, and behavioral components)
Emergency medicine crisis resource management (EMCRM)	Wallin et al. (2007)	Team coordination skills (anticipation of potential problems, asking for help, attention allocation, communication, delegation, knowledge of the environment, leadership, relational capabilities, teamworking and team leadership, use of information and resources)
Emotional Intelligence Training Program	Gilar-Corbí et al. (2018)	Adaptability and decision-making (impulse control, problem solving); emotion management; interpersonal skills (empathy, relationship management, social responsibility); mood and self-expression (emotional expression, assertiveness, independence); self-perception (self-awareness, self-esteem, self-realization); stress management (flexibility, stress tolerance, optimism)
Executive Leadership and Organization Change (ELOC) program	Rhee and Sigler (2010)	Change catalyst; conflict management; leadership; system thinking; service orientation; team management
Girls First Resilience Curriculum	Leventhal et al. (2015)	Adaptability; coping capabilities; emotional resilience; persistence; self-efficacy; social skills
Happy 12–16	Filella et al. (2018)	Conflict resolution; self-control and emotion regulation
Leadership Assessment and Development (LEAD) course	Boyatzis et al. (2002); Rhee (2008); Camuffo et al. (2009); Passarelli et al. (2018)	Achievement orientation; adaptability; coaching and mentoring; conflict management; empathy; goal setting; influence; information analysis; information gathering; initiative; inspirational leadership; organizational awareness; pattern recognition; positive outlook; self-awareness; self-control; system thinking; teamwork
MindOut social and emotional learning program	Dowling et al. (2019)	Relationship management; responsible decision-making; self-awareness; self-management; social awareness
PREDEMA	Schoeps et al. (2018)	Expression of emotions; self-awareness; self-control and emotion regulation; social awareness
PREDEMA 2.0	Schoeps et al. (2019)	Assertiveness; conflict resolution; empathy; positive mood; self-awareness; self-control and emotion regulation; self-esteem; social awareness
Professional Fellows program	Ballou et al. (1999)	Attention to detail; communication; developing others; efficiency orientation; empathy; flexibility; group management; initiative; negotiation; networking; pattern recognition; persuasiveness; planning; quantitative analysis; self-confidence; self-control; social objectivity; systems thinking; use of concepts
ReSource Project	Trautwein et al. (2020)	Attention; awareness; socio-affective skills (i.e., compassion, prosocial motivation); socio-cognitive skills (i.e., perspective taking, understanding others)
Reflective team case teaching model	Griffith (1999)	Cognitive intelligence skills (critical thinking); emotional intelligence skills (communication, perspective taking, teamworking)
The Cleveland Clinic Academy curriculum for health care leadership development	Stoller (2013)	Leadership competencies
Tuning into Teens	Havighurst et al. (2015)	Emotion coaching (conflict resolution, empathy, management of others’ emotions); self-awareness; self-control and emotion regulation
Whole person learning approach	Hoover et al. (2010); Barbera et al. (2015)	Communication; decision-making (negotiation, conflict management, and resolution); leadership; planning; self-awareness; social awareness; teamwork

When considering the most targeted competencies, literature reveals a predominant focus on emotional self-awareness (Boyatzis et al., 2002; Mirvis, 2008; Rhee, 2008; Hoover et al., 2010; Barbera et al., 2015; Harris et al., 2016; Gilar-Corbí et al., 2018; Passarelli et al., 2018; Schoeps et al., 2018, 2019; Dowling et al., 2019; Jennings et al., 2019; Trautwein et al., 2020), namely the ability to understanding one’s own emotions and their effects. The LEAD course, the whole person learning approach, and other programs that aim to promote learners’ wellbeing, such as the MindOut social and emotional learning program, CARE for teachers, or the CALM course, are structured in ways that allow a deeper reflection on learners’ emotions, reactions, desires, and values (Boyatzis et al., 2002; Rhee, 2008; Harris et al., 2016; Passarelli et al., 2018; Dowling et al., 2019; Jennings et al., 2019).

The second most developed behavioral competency is self-control (Ballou et al., 1999; Boyatzis et al., 2002; Rhee, 2008; Taylor and Boyatzis, 2012; Havighurst et al., 2015; Harris et al., 2016; Filella et al., 2018; Gilar-Corbí et al., 2018; Passarelli et al., 2018; Schoeps et al., 2018, 2019; Dowling et al., 2019; Jennings et al., 2019), followed by social awareness competencies like empathy (Ballou et al., 1999; Boyatzis et al., 2002; Wallin et al., 2007; Mirvis, 2008; Rhee, 2008; Hoover et al., 2010; Barbera et al., 2015; Havighurst et al., 2015; Gilar-Corbí et al., 2018; Passarelli et al., 2018; Schoeps et al., 2018, 2019; Dowling et al., 2019; Trautwein et al., 2020). Being able to manage ourselves even during the most stressful events has important implications for the quality of our social relationships, and it has an impact on our performance. When we lose control of our emotions, we are subjected more to feelings and thoughts that nourish our negative states, impeding a lucid evaluation of situations and negatively affecting our ability to manage and solve problems. Moreover, people who demonstrate the ability to understand other people’s emotions deeply and ‘step into others’ shoes’ are more inclined to collaborate with others, be good team workers and leaders and, more generally, they can reach a superior performance thanks to their ability to ‘harmonize’ with people with which they interact (Goleman, 1998).

Taken together, these results suggest that knowledge of our emotions, along with a deep understanding of others’ feelings and the ability to act and react in accordance with our own and others’ emotional states, is the foundation of most of the development programs considered in this review. Indeed, if we consider the educational programs based on MBIs, they are often devoted to nurturing self-awareness in combination with emotion regulation/stress reduction, empathy, and compassion (Trautwein et al., 2020).

Another example where these competencies are jointly developed is offered by the Tuning into Teens program (Havighurst et al., 2015). This is a modified version of the Tuning into Kids program, adapted to address adolescents’ needs. Both programs aim at training parents to recognize and respond to their children’s emotions in order to promote and support emotional awareness and regulation in youths. To accomplish this goal, the Tuning into Teens and Tuning into Kids programs also promote parents’ emotional self-awareness and their ability to manage their emotions. These programs are designed according to the model of emotion coaching proposed by Gottman and DeClaire (1997) that condenses into five suggestions: (1) be aware of your children’s emotions, especially when they are of low intensity; (2) recognize the importance of emotions for establishing deep contact with your children; (3) be empathetic with your children and communicate to them that they are understood; (4) support children in communicating, describing, and labeling their feelings; and (5) support and help children in solving problems in relation to their emotions, if needed, but leave room for self-improvement and autonomy (Havighurst et al., 2009).

Social and emotional self-awareness represent the premise for successful development of other behavioral competencies (Goleman, 1998), like those most frequently addressed in other studies, such as conflict management (Rhee, 2008; Rhee and Sigler, 2010; Havighurst et al., 2015; Filella et al., 2018; Passarelli et al., 2018; Schoeps et al., 2019) and leadership (Boyatzis et al., 2002; Rhee, 2008; Hoover et al., 2010; Rhee and Sigler, 2010; Stoller, 2013; Barbera et al., 2015; Passarelli et al., 2018). The ability to manage difficult situations, encouraging an open dialogue about problems and finding creative solutions to possible frictions, is at the heart of success in many roles and positions. It is fundamental especially for activities that require many interactions, and for people working under pressure (Goleman, 1998). It is also a fundamental skill that outstanding leaders should manifest. Effective leaders are able to be at the forefront of any project/activity with enthusiasm and energy. They are able to motivate their followers, being a model of conduct, but also deeply understanding others’ emotions and the reasons behind their behaviors.

### The Effectiveness of the Competency Development Programs

This section aims to shed light on the effectiveness of those educational programs that are designed for both the direct and the indirect development of behavioral competencies. We present the results of our analysis as follow: the first section focuses on the methodology adopted by the authors to test the effectiveness of the analyzed educational programs. The second paragraph explores the determinants of the development of emotional and social intelligence competencies, specifically considering both the learners’ educational experience and their personal characteristics. Finally, we present the effects of these educational programs on other variables, particularly on participants’ entrepreneurial intention, wellbeing, academic performance, and career-related variables.

#### Methodology Adopted for Assessing Behavioral Competency Development

An analysis of the methodologies employed in the empirical studies (48 out of 71 articles) that assess the impact of the aforementioned programs allows us to highlight some peculiarities. Most of the studies directly targeting competency development adopt an experimental design (42%). In these works, participants are randomly divided into two groups, the experimental and the control group. The first receives the treatment, participating in a competency development program, while the second does not attend any course. Competencies are measured in both groups before (at t_1_) and after (at t_2_) the experiment. This procedure is adopted in studies such as those by Bücker and Korzilius (2015), Gilar-Corbí et al. (2018), Dowling et al. (2019), and other scholars (Havighurst et al., 2015; Leventhal et al., 2015; Harris et al., 2016; Jennings et al., 2019). A quarter of the empirical articles assessing programs specifically targeting ESC competency development are further characterized by a quasi-experimental design. The experimental and quasi-experimental procedures differ in the assignment of participants to experimental and control groups, which is random in the first case. Ultimately, the procedure for assessing the improvement of behavioral competencies in the intervention group after the course is similar to that described when presenting the experimental approach (see, for instance, Hoover et al., 2010; Ramsey and Lorenz, 2016; Filella et al., 2018; Schoeps et al., 2019). The work by Schoeps et al. (2018), however, is noteworthy. The authors collected measures about the effects of the intervention program at three different points in time: before the experiment, just at the end of the protocol, and 6 months post-test, in order to measure the long-term effect of the program.

Ten studies in our dataset adopt a longitudinal design (Boyatzis et al., 2002; Leonard, 2008; Wheeler, 2008; Camuffo et al., 2009; Amdurer et al., 2014; Gong et al., 2016; McGue et al., 2017; Passarelli et al., 2018; Dave et al., 2019; Oi, 2019). In these works, authors assess the long-term impact of each development program and discuss its effectiveness in light of a sustainable development. More details are provided in Table 4 which displays the characteristics of the research conducted in the quantitative studies in our data set.

**TABLE 4:** Research design of the quantitative studies on competency development.

Study	Study design	Sample size	Target audience	Target behavioral competencies	Developed behavioral competencies	Pre–post-test	Long-term follow-up	Self/other assessment
Amdurer et al. (2014)	Longitudinal cohort study	*N* = 266	Graduate students of an MBA program at the Weatherhead School of Management (WSOM), Case Western Reserve University (Cleveland, OH, United States).	Attention to detail, developing others, efficiency orientation, emotional self-control, empathy, flexibility, group management, negotiating, networking, oral communication, pattern recognition, persuasiveness, planning, quantitative analysis, self-confidence, social objectivity, system thinking, use-of-concepts, use- of-technology, written communication.	Attention to detail, developing others, efficiency orientation, emotional self-control, empathy, flexibility, group management, negotiating, networking, oral communication, pattern recognition, persuasiveness, planning, quantitative analysis, self-confidence, social objectivity, system thinking, use-of-concepts, use- of-technology, written communication.	Pre–post-test assessment.	–	Self-assessment, external raters and third-party evaluation.
Arco-Tirado et al. (2011)	Experiment	*N* = 141	Freshmen and tutors from the University of Granada (Spain).	Social skills [ability of saying ‘no,’ make requests, initiate positive interactions with the opposite sex, defense of own interests, expression of discomfort, self-expression].	Social skills [ability of saying ‘no,’ make requests, initiate positive interactions with the opposite sex, defense of own interests, expression of discomfort, self-expression].	Pre–post-test assessment.	–	Self-assessment.
Ballou et al. (1999)	Mixed	*N* = 53	People enrolled in the Professional Fellows Program.	Attention to detail, developing others, efficiency orientation, empathy, flexibility, group management, initiative, negotiating, networking, oral communication, pattern recognition, persuasiveness, planning, quantitative analysis, self-confidence, self-control, social objectivity, systems thinking, theory building, use of concepts, using technology, written communication.	Developing others, efficiency orientation, empathy, flexibility, group management, initiative, networking, oral communication, pattern recognition, persuasiveness, planning, self-confidence, self-control, systems thinking, use of concepts.	Pre–post-test assessment.	–	Self-assessment, external raters and third-party evaluation.
Bonesso et al. (2015)	Correlational study	*N* = 95	First year university students enrolled in master’s degree at an Italian university.	Emotional competencies [accurate self-assessment, achievement orientation, adaptability, conflict management, conscientiousness, emotional awareness, emotional self-control, initiative, optimism, self-confidence, trustworthiness], Social competencies [building bonds, change catalyst, communication, cultural awareness, developing others, empathy, influence, inspirational leadership, organizational awareness, service orientation, teamwork], Cognitive competencies [pattern recognition, systems thinking].	IEL methods are particularly effective for developing self-awareness and self-management. SEL and TL methods are particularly effective for developing Social competencies; IEL and SEL techniques are particularly effective for developing Cognitive competencies.	Post-test assessment	–	Self-assessment and external raters.
Bonesso et al. (2018)	Causal comparative study	*N* = 142	Students from an Italian university who were close to graduate or recently graduated.	Emotional competencies [achievement orientation, adaptability, conscientiousness, emotional self-awareness, emotional self-control, positive outlook], Social competencies [change catalyst, coach and mentor, conflict management, empathy, influence, inspirational leadership, organizational awareness, teamwork], Cognitive competencies [pattern recognition, systems thinking].	International Experience positively influence all group of competencies. Cultural Experience positively influences Social and Cognitive competencies.	Post-test assessment.	–	External raters.
Boyatzis et al. (2002)	Longitudinal cohort study	*N* = 1241	MBA students at the Weatherhead School of Management, Case Western Reserve University (Cleveland, OH, United States).	Relationship-management [developing others, empathy, group management, helping, leadership, negotiating, networking, oral communication, persuasiveness, relationship, social objectivity]. Cognitive competencies [information analysis, information-gathering, pattern recognition, quantitative analysis, sense-making, systems-thinking, theory-building, use-of-concepts, use-of-technology, written communication]. Self-management competencies [action, attention-to-detail, efficiency orientation, flexibility, goal setting, initiative, planning, self-confidence, self-control].	Relationship-management [developing others, empathy, group management, helping, leadership, negotiating, networking, oral communication, persuasiveness, relationship, social objectivity]. Cognitive competencies [information analysis, information-gathering, pattern recognition, quantitative analysis, sense-making, systems-thinking, theory-building, use-of-concepts, use-of-technology, written communication]. Self-management competencies [action, attention-to-detail, efficiency orientation, flexibility, goal setting, initiative, planning, self-confidence, self-control].	Pre–post-test assessment.	–	Self-assessment, external raters and third-party evaluation.
Bücker and Korzilius (2015)	Quasi-experiment	*N* = 81.	University students studying International Management.	Cultural intelligence (behavioral, cognitive, metacognitive, motivational).	Cultural intelligence (behavioral, metacognitive, motivational).	Pre–post-test assessment.	–	Self-assessment.
Burch et al. (2016)	Causal comparative study	*N* = 180	Undergraduate business students at a Southern U.S. university.	Cognitive competencies [analytical thinking, knowledge application, reflective thinking], Social competencies [social awareness, relationship management].	Students in face-to-face classes develop their Cognitive competencies [analytical thinking, knowledge application, reflective thinking] and Social competencies [social awareness, relationship management] better than students in web-based classes.	Post-test assessment.	–	Self-assessment.
Camuffo et al. (2009)	Longitudinal cohort study	*N* = 44	Graduates of an MBA program held at the Fondazione CUOA (Italy).	Attention to detail, business bargaining, customer orientation, developing others, directing others, efficiency orientation, empathy, flexibility, group management, information gathering, initiative, leadership, negotiating, networking, oral communication, organizational awareness, organizational commitment, pattern recognition, persuasiveness, planning, result orientation, quantitative analysis, self-confidence, self-control, social objectivity, system thinking, theory building, use of concepts, use of technology, visioning, written communication.	Directing others, efficiency orientation, networking, written communication.	Pre–post-test assessment.	–	Self-assessment, external raters and third-party evaluation.
Cronin et al. (2019)	Cross-sectional study	*N* = 407	Physical Education students from five schools in England and one school in Ireland.	Life skills [decision making, emotional skills, goal setting, interpersonal communication, leadership, problem solving, social skills, teamwork, time management].	Life skills [decision making, emotional skills, goal setting, interpersonal communication, leadership, problem solving, social skills, teamwork, time management].	Students completed the survey during physical education lessons or at the beginning of the school day.	–	Self-assessment.
Dave et al. (2019)	Longitudinal study	*N* = 1.400	Canadian children and youths.	Emotional Intelligence related competencies [interpersonal skills, intrapersonal skills, stress management, and adaptability].	Emotional Intelligence related competencies [interpersonal skills, intrapersonal skills, stress management, and adaptability].	The study uses data collected by the National Longitudinal Survey of Children and Youth starting from 2004.	Follow-ups were conducted as part of the National Longitudinal Survey in 2006 and 2008.	Self-assessment.
Dowling et al. (2019)	Experiment	*N* = 497	Students in schools for youth from disadvantaged communities.	Relationship management, responsible decision-making, self-awareness, self-management, social awareness.	Self-management.	Pre–post-test assessment.	–	Self-assessment.
Farashahi and Tajeddin (2018)	Correlational study	*N* = 194	Undergraduate and MBA students from a Canadian business school.	Interpersonal skills, problem solving skills, self-awareness.	Simulation are effective for developing interpersonal competencies and self-awareness, followed by case study and lecture, respectively. Simulations and case studies are particularly effective for developing problem solving skills.	Post-test assessment.	–	Self-assessment.
Fernández-Pérez et al. (2019)	Quasi-experiment	*N* = 862	University students at the University of Granada (Spain).	Empathy, motivation, self-awareness, self-regulation, social skills-leadership.	Empathy, motivation, self-awareness, self-regulation, social skills-leadership.	Pre–post-test assessment.	–	Self-assessment.
Filella et al. (2018)	Quasi-experiment	*N* = 903	Spanish adolescents enrolled in secondary education programs.	Conflict resolution, emotion regulation, self-control.	Emotion regulation, self-control.	Pre–post-test assessment.	–	Self-assessment.
Gilar-Corbí et al. (2018)	Experiment	*N* = 192	University students.	Adaptability and decision-making [impulse control, problem solving], emotion management, interpersonal skills [empathy, relationship management, social responsibility], mood and self-expression [emotional expression, assertiveness, independence], self-perception [self-awareness, self-esteem, self-realization], stress management [flexibility, stress tolerance, optimism].	Adaptability and decision-making [impulse control, problem solving], emotion management, interpersonal skills [empathy, relationship management, social responsibility], mood and self-expression [emotional expression, assertiveness, independence], self-perception [self-awareness, self-esteem, self-realization], stress management [flexibility, stress tolerance, optimism].	Pre–post-test assessment.	–	Self-assessment.
Gong et al. (2016)	Longitudinal study	*N* = 1.795	Chinese rural children.	Attention skills [cognitive self-control, concentration, discipline, organization], Social skills [sociability/number of friends, leadership at school].	Social skills [sociability/number of friends, leadership at school].	The study uses data from the China Family Panel Studies. Data were collected starting from 2010.	This study uses the follow-up conducted in 2012.	Self-assessment.
Harris et al. (2016)	Experiment	*N* = 64	Educators in two middle schools.	Positive emotions (compassion, acceptance), self-awareness, self-control and emotion regulation, sense of community.	Self-control and emotion regulation.	Pre–post-test assessment.	–	Self-assessment.
Havighurst et al. (2015)	Experiment	*N* = 449	Parents and their adolescent offspring.	Emotion coaching (conflict resolution, empathy, management of others’ emotions), self-awareness, self-control and emotion regulation.	Emotion coaching (conflict resolution, empathy, management of others’ emotions), self-awareness, self-control and emotion regulation.	Pre–post-test assessment.	–	Self-assessment and external raters.
Hoover et al. (2010)	Quasi-experiment	*N* = 483	MBA students who completed a course in executive skills.	Communication, decision-making [negotiation, conflict management, conflict resolution], leadership, planning, self-awareness, social awareness, teamwork.	Communication, decision-making [negotiation, conflict management, conflict resolution], leadership, planning, self-awareness, social awareness,	Pre–post-test assessment.	–	Third-party evaluation.
Jennings et al. (2019)	Experiment	*N* = 224	Elementary school teachers in high poverty areas of New York.	Emotional support, self-awareness, self-control and emotion regulation.	Emotional support, self-awareness, self-control and emotion regulation.	Pre–post-test assessment.	–	Self-assessment and external raters.
Leonard (2008)	Longitudinal study	*N* = 100	Graduates of an MBA program.	Attention to detail, developing others, efficiency orientation, emotional self-control, empathy, flexibility, group management, initiative, negotiating, networking, pattern recognition, persuasiveness, planning, self-confidence, social objectivity, system thinking.	Attention to detail, developing others, efficiency orientation, emotional self-control, empathy, flexibility, group management, initiative, negotiating, networking, pattern recognition, persuasiveness, planning, self-confidence, social objectivity, system thinking.	Pre–post-test assessment.	–	Self-assessment, and third-party evaluation.
Leventhal et al. (2015)	Experiment	*N* = 2.387	Rural adolescent girls in Bihar (India).	Adaptability, coping capabilities, emotional resilience, persistence, self-efficacy, social skills.	Social skills.	Pre–post-test assessment.	–	Self-assessment.
McGue et al. (2017)	Longitudinal study	Two studies: the Minnesota Twin Family Study: *N* = 1.382; the Sibling Interaction and Behavior Study: *N* = 617.	The Minnesota Twin Family Study (MTFS): pairs of twins and their parents; The Sibling Interaction and Behavior Study (SIBS): adoptive and nonadoptive families with two offspring and their rearing parents.	Non-Cognitive skills [academic effort, academic problems, aggression, alienation, control, externalizing, harm avoidance, stress reaction].	–	MTFS: twins are assessed at 11 or 17 years old; SIBS: adolescents were assessed at the mean age of 15 years old.	MTFS: follow-ups were conducted at the age of 14, 17, 20, 24, 29; SIBS: follow-ups were conducted after 3, 7 years.	Self-assessment.
Mendo-Lázaro et al. (2018)	Quasi-experiment	*N* = 346	University students from the Degrees in Infant Education and Primary Education at the University of Extremadura (Spain).	Social skills: (1) self-assertion [asking for changes in behavior, taking criticism on board, stopping interaction], (2) receiving information [actively listening, empathizing, summarizing, asking for help, asking questions], (3) imparting information [motivating, imparting information, convincing others, explaining oneself, giving help].	Imparting information [motivating, imparting information, convincing the others, explaining oneself or helping], self-assertion [asking for changes in behavior, receiving criticism).	Pre–post-test assessment.	–	Self-assessment.
Oi (2019)	Longitudinal study	*N* = 9.291	U.S. individuals from adolescence to adulthood.	Non-cognitive skills [risk aversion, future-orientation, and conscientiousness].	Non-cognitive skills [risk aversion, future-orientation, and conscientiousness].	The study uses data collected by the National Longitudinal Study of Adolescent to Adult Health during 1994.	Follow-ups were conducted in 1995, 2001, 2008.	Self-assessment.
Passarelli et al. (2018)	Longitudinal cohort study	*N* = 531	Full-time graduate MBA students.	Achievement orientation, adaptability, coach and mentor, conflict management, emotional self-awareness, emotional self-control, empathy, influence, inspirational leadership, organizational awareness, pattern recognition, positive outlook, teamwork, system thinking.	Achievement orientation, adaptability, coach and mentor, emotional self-awareness, empathy, influence, inspirational leadership, pattern recognition, system thinking.	Pre–post-test assessment.	–	Self-assessment and external raters.
Ramsey and Lorenz (2016)	Quasi-experiment	*N* = 281	U.S graduate students in business subjects.	Cultural intelligence (cognitive, metacognitive, motivational, and behavioral).	Cultural intelligence (cognitive, metacognitive, motivational, and behavioral).	Pre–post-test assessment.	–	Self-assessment.
Rhee (2008)	Mixed	*N* = 25	MBA students who attended the LEAD course in an MBA program at the Weatherhead School of Management, Case Western Reserve University (Cleveland, OH, United States).	Action, goal setting, help, information analysis, information gathering, initiative, leadership, quantitative analysis, relationship building, sense making, theory building, use of technology.	Action, goal setting, help, information analysis, information gathering, initiative, leadership, quantitative analysis, sense making, theory building, use of technology.	Pre–post-test assessment.	–	Self-assessment and third-party evaluation.
Riggs et al. (2010)	Correlational study	Study 1: *N* = 46; Study 2: *N* = 118	Study 1: Adolescents in low-income status; Study 2: Latino students with poor academic performance, poor classroom conduct and low parent involvement in school.	Study 1: self-worth; Study 2: concentration and emotional regulation skills.	Study 1: self-worth; Study 2: concentration and emotional regulation skills, only for individuals with preexisting concentration and emotional regulation problems.	Pre–post-test assessment.	–	Self-assessment and external raters.
Ruiz-Ariza et al. (2018)	Experiment	*N* = 190	Adolescents enrolled at two summer schools in Andalusia (Spain).	Emotional intelligence [self-control, emotionality, sociability].	Emotional intelligence [sociability].	Pre–post-test assessment.	–	Self-assessment.
Schoeps et al. (2018)	Quasi-experiment	*N* = 148	Adolescents enrolled in first and second years of secondary school.	Expression of emotions, self-awareness, self-control and emotion regulation, social awareness.	Expression of emotions, self-awareness, self-control and emotion regulation, social awareness, not immediately after the intervention but after the 6 months follow-up.	Pre–post-test assessment.	A follow-up was conducted 6 months after the end of the intervention.	Self-assessment.
Schoeps et al. (2019)	Quasi-experiment	*N* = 250	Undergraduate students enrolled at a Spanish university.	Assertiveness, conflict resolution, empathy, positive mood, self-awareness, self-control and emotion regulation, self-esteem, social awareness.	–	Pre–post-test assessment.	A follow-up was conducted 3 months after the end of the intervention.	Self-assessment.
Sheehan et al. (2009)	Quasi-experiment	*N* = 49	Undergraduate and graduate students.	Emotional and Social competencies [achievement orientation, accurate self-assessment, adaptability, building bonds, change catalyst, communication, conflict management, cultural awareness, developing others, emotional self-awareness, emotional self-control, empathy, influence, initiative, inspirational leadership, optimism, organizational awareness, self-confidence, service orientation, teamwork and collaboration, transparency, trustworthiness].	Emotional and Social competencies: [accurate self-assessment, achievement orientation, trustworthiness, adaptability, optimism, service orientation, organizational awareness, cultural awareness, communication, building bonds, and teamwork and collaboration].	Post-test assessment.	No follow-ups.	External raters and third-party evaluation.
Thompson et al. (2019)	Cross-comparative study	*N* = 113	MBA students, psychology students, nonstudents [control groups].	Emotional Intelligence.	Emotional Intelligence only for MBA students.	Pre–post-test assessment.	–	Self-assessment.
Trautwein et al. (2020)	Experiment	*N* = 332	Healthy adults.	Attention, awareness, socio-affective skills (compassion, prosocial motivation), socio-cognitive skills (perspective taking, understanding others).	Attention, compassion.	Pre–post-test assessment.	–	Third-party evaluation.
Turner and Mulholland (2017)	Correlational study	*N* = 117	Business Studies students from secondary schools and one university in Dundee (Scotland, United Kingdom).	Entrepreneurial skills [collaboration, communication, confidence, creativity and innovation, decision-making, leadership, organization and management, problem-solving, resolving conflict, reflection].	Entrepreneurial skills [communication, confidence, creativity and innovation, organization and management, reflection].	Post-test assessment.	–	Self-assessment.
Wallin et al. (2007)	Experiment	*N* = 15	Medical students at the beginning of their clinical career.	Team coordination skills [anticipation of potential problems, asking for help, attention allocation, communication, delegation, knowledge of the environment, leadership, relational capabilities, teamworking and team leadership, use of information and resources].	Team coordination skills [anticipation of potential problems, asking for help, attention allocation, communication, delegation, knowledge of the environment, leadership, relational capabilities, teamworking, use of information and resources].	Pre–post-test assessment.	–	Self-assessment and third-party evaluation.
Wheeler (2008)	Longitudinal study	*N* = 30	Part-time MBA students.	Emotional competencies [attention to detail, efficiency orientation, flexibility, initiative, planning, self-control], Social competencies [developing others, empathy, group management, leadership, negotiating, networking, persuasiveness, self-confidence], Cognitive competencies [pattern thinking, social objectivity, systems thinking].	Emotional competencies [efficiency orientation, flexibility, initiative, self-control], Social competencies [leadership, self-confidence], Cognitive competencies [social objectivity, systems thinking].	Pre–post-test assessment.	A follow-up was conducted between 18 and 30 months after the graduation.	Self-assessment and third-party evaluation.

This comparative analysis report information about the research design, the sample, the assessment procedure (pre-posttest assessment, follow-ups, actors involved), along with the targeted and developed behavioral competencies. As for this last aspect, a number of studies reveals their effectiveness for the development of a wide range of skills, such as the emotional, social and cognitive intelligence competencies (see for instance Ballou et al., 1999; Rhee, 2008; Sheehan et al., 2009; Bonesso et al., 2015, 2018; Gilar-Corbí et al., 2018; Passarelli et al., 2018), while other research narrows the focus to more specific clusters related, for instance, to individuals’ social sphere and relationship management (e.g., Arco-Tirado et al., 2011; Mendo-Lázaro et al., 2018).

Some differences in the methods adopted for measuring the effects of the learning experience on behavioral competencies were found in those programs not explicitly designed for developing ESCs. The majority (53%) of articles in this last group lean toward a quantitative design, collecting cross-sectional data. This is the case, for instance, of Burch et al.’s (2016) article, in which the authors rely on a survey design to compare the unintended outcomes of web-based and face-to-face classes. Questionnaires were also administrated by Farashahi and Tajeddin (2018) to compare the effects of three teaching procedures, and by Bonesso et al. (2015), who assess the joint impact of different learning methods on students’ ESC competencies. Moreover, in this last group of works, only two articles (Arco-Tirado et al., 2011; Mendo-Lázaro et al., 2018) adopt, respectively, an experimental and a quasi-experimental design, further highlighting the differences between the two aforementioned groups of programs.

#### Factors Influencing the Development of Behavioral Competencies

Along with the courses that proved their effectiveness for developing ESCs, literature has identified other intervening factors that may enhance or hinder the effectiveness of the learning process. We categorized these variables into two broad categories: the one referring to characteristics of the learning experience, while the other to learners’ personal characteristics.

For what concerns the former category, literature has acknowledged the importance of complementing traditional learning methods, such as lectures, with other, more holistic and experience-based, approaches. In this regard, Bonesso et al. (2015) showed that emotional and social competencies can be effectively developed in students only when individual and social experiential learning methods (i.e., simulations, individual/cooperative assignments, business games) are integrated into the more traditional educational activities. Similar results are provided by Farashahi and Tajeddin (2018) who tested the effectiveness of three different teaching methods aimed at enhancing learners’ self-awareness, interpersonal and problem-solving skills. The authors confirm that simulations are perceived as the most effective teaching method for developing relational capabilities and self-awareness. Case study approach is perceived as effective as simulations for stimulating problem solving skills, while it is the second-best method for developing interpersonal competencies and self-awareness. More interestingly, students classify lectures as the less effective approach for their behavioral competency development (Farashahi and Tajeddin, 2018). To further corroborate these results and moving from the in-class environment, Bonesso et al. (2018) suggest that living an international experience and taking part to cultural activities favor the development of behavioral competencies. When abroad, students get in touch with cultures, uses and norms that detach them from the comforting familiarity of their habits. They experience aloneness and need to construct new relationships to cope with it and to integrate in their new social environment. Similarly, when taking part to cultural activities (e.g., political parties, artistic groups, volunteerism) individuals interact and cooperate with others, often facing situations that ask them to reflect on, and sometimes revise, their values and ethical beliefs. These experiences proved to be of stimulus for the development of students’ emotional, social and cognitive competencies.

As for the category grouping learners’ personal features, the work by Cronin et al. (2019) confirms that students’ basic needs satisfaction acts as a mediator between the teaching style of their coach and the development of their life skills (i.e., interpersonal communication, emotional capabilities, leadership skills, problem solving competencies). The authors suggest that a teaching style oriented to encouraging students’ proactivity and initiative and that promotes their problem-solving and interpersonal skills has the effect of fulfilling learners’ need for autonomy and relatedness while enhancing their behavioral competency development. In studying the process of change that underlies the development of behavioral competencies, Rhee (2008) discovered the key role played by students’ career aspirations, opportunity mode and desire for continuous learning. The author suggests that individuals who possess a higher propensity to continuous learning, who have planned a clear career path and who try to exploit their learning opportunity in order to maximize their learning outcomes obtain better results in term of competency development compared to students who do not show these characteristics. Finally, in a work reaffirming the importance of having a clear and structured learning plan that guide the acquisition of behavioral competencies, Leonard (2008) provides support to the hypothesis that individuals who have a clear image of who they want to become and of the competencies they need to improve are more successful in their process of change than those who do not establish clear learning goals.

#### The Effectiveness of Competency Development Programs on Other Variables

Among the reviewed programs, we identified a group of studies that not only address the enhancement of behavioral skills *per se*, but analyze the effect of competency development on other variables. Table 5 reports the description of the dependent variables that have been affected by the behavioral competencies developed by the educational programs.

**TABLE 5:** The impact of competency development on other variables.

Study	Dependent variables
Amdurer et al. (2014)	Life satisfaction, career satisfaction, and career success: self-informant based assessment of validated measures.
Bonesso et al. (2018)	Entrepreneurial orientation measured as the expression of a conscious plan to set up a business.
Camuffo et al. (2009)	Career advancement. Two measures: (i) position held by each subject (classified according to an adapted version of the Watson Wyatt Global Grading System and weighted considering the information related to the subject’s position in the company hierarchy and the number of collaborators that he/she manages); (ii) the characteristics of the company which employed the subject at the moments under examination (in terms of turnover, number of employees, organizational complexity). Career compensation: total annual wage (fixed and variable) of each subject.
Fernández-Pérez et al. (2019)	Entrepreneurial intention: self-informant based assessment of a validated measure.
Harris et al. (2016)	Positive affect, distress tolerance, teaching efficacy, professional, burnout, physical symptoms: self-informant based assessment of validated measures. Blood pressure readings. Diurnal assays of cortisol.
Jennings et al. (2019)	Psychological distress and physical distress: self-report and report on student assessments of validated measures. Emotional support: classroom observation on teacher–student interactions.
Leventhal et al. (2015)	Positive psychological wellbeing: self-informant based assessment with the KIDSCREEN-Psychological Wellbeing Subscale. Social wellbeing: with the KIDSCREEN-Social Support and Peers Subscale
McGue et al. (2017)	College outcome: measured as having completed or currently attending college at the second follow-up.
Schoeps et al. (2018)	Cyberbullying, life satisfaction: self-informant based assessment of a validated measures of before (T1), immediately after (T2), and 6 months after the intervention (T3).
Schoeps et al. (2019)	Subjective well-being: self-informant assessment of a validated scale of life satisfaction.

Bonesso et al. (2018) and Fernández-Pérez et al. (2019) test and confirm the effectiveness of ESC competency development on students’ entrepreneurial intention. Entrepreneurial intention is defined as a planned desire to start a new business in the future (Bonesso et al., 2018). Persons who possess or develop their entrepreneurial intention are more likely to show entrepreneurial behaviors that concretize into the creation of new businesses (Fernández-Pérez et al., 2019). In particular, it has been discovered that students who receive entrepreneurial training and develop their emotional competencies during these courses show higher entrepreneurial intention, since they develop their entrepreneurial attitude and self-efficacy. Emotional competencies in fact increase students’ self-identification as future entrepreneurs and strengthen confidence in their ability as future business owners (Fernández-Pérez et al., 2019). Moreover, learning how better to understand and manage themselves and others’ emotions has a direct impact on learners’ entrepreneurial intention (Bonesso et al., 2018).

Some studies have considered the impact of competency development on improving people’s wellbeing. This is the case of the PREDEMA and PREDEMA 2.0 programs (Schoeps et al., 2018, 2019). The authors assert that intervening in students’ behavioral competencies helps them cope with the pressures associated with academic life, especially during the transition from high school to the university (Schoeps et al., 2019), while it also shows positive effects in improving the coexistence between adolescents in classroom environments, limiting the incidence of cyberbullying behaviors (Schoeps et al., 2018). Similar findings are reported in a study on the efficacy of the Girls First Resilience Curriculum, designed and implemented for girls in high-poverty conditions (Leventhal et al., 2015). The program reveals its effectiveness in enhancing girls’ psychological and social wellbeing, meaning that people enrolled in this curriculum show, at the end of the course, higher levels of life satisfaction and greater capacity to build relationships with peers. These results are obtained through the improvement of behavioral competencies such as self-efficacy, tolerance of negative situations, empathy, and teamworking (Leventhal et al., 2015).

As mentioned earlier, teachers are responsible not only for the quality of education they provide to their students, but also for students’ development as human beings. Promoting teachers’ wellbeing and their capacity to manage stressful situations is at the heart of CARE for teachers and CALM programs. These MBIs reveal their effectiveness in improving educators’ ESC competencies and in promoting participants’ wellbeing and distress, with positive results also in terms of educational outcomes (Harris et al., 2016; Jennings et al., 2019).

Some studies have tested the impact of the development of behavioral competencies on learners’ educational attainments and career-related variables such as career satisfaction and career success. According to McGue et al. (2017), cognitive and non-cognitive competencies, especially self-control, emotion control, and motivation, are independently associated with students’ academic achievements. Thus, students who are able to manage their negative emotions even in the toughest periods are more inclined to achieve challenging objectives in the long run instead of pursuing immediate reward. Another explanation comes from a literature review conducted by Waters et al. (2014), in which the authors contend that MBIs increase learners’ attention, memory, and emotion regulation. Meditation is able to change learners’ brains and to foster learners’ cognitive functions and self-control, supporting their motivation and the creation of a personalized learning plan, thus leading them toward successful academic outcomes, greater wellbeing, and better social relationships (Waters et al., 2014).

As for career-related variables, the literature suggests that the development of emotional intelligence competencies positively impacts on career satisfaction and career success, used as umbrella terms for capturing one’s own idea of satisfaction with one’s position, income level, advancement chances, and development opportunities inside the organization (Amdurer et al., 2014). More specifically, a study by Camuffo et al. (2009) reveals that students who participated in an MBA program and who reported higher improvements (measured by self-, peer, and third party evaluations) in their ESC competencies during the course, have better career paths (measured by a composite index considering the subjects’ position, the number of collaborators, and the characteristics of the organization) at least 1 year after the end of the MBA program.

## Literature Gaps and Directions for Future Research

There is little doubt that the debate on how emotional, social, and cognitive competencies should be learnt has flourished during the last decade, with contributions in the literature that document the educational programs designed to nurture different behaviors in diverse learning contexts. The results of our analysis also allow the identification of major gaps in the current debate and promising areas for future research.

### Categories of Learner: Who Is Missing?

The review reveals that competency development programs have been designed and delivered in different settings, from high school to higher education and organizational contexts. The studies claim a positive influence of ESC programs in supporting individuals toward transition stages in their educational and career paths. However, where the higher education setting is concerned, the ESC learning experience offered in Bachelor, graduate, or Master’s programs are often attended only by students enrolled in management curricula. Research has shown that behavioral competencies are crucial in every job and every sector; therefore, their development does not pertain only to professional roles operating in the management field. Consequently, further studies should consider the design of ESC courses in different disciplinary areas, focusing on those competencies most required in the labor market and distinctive for the specific professional roles prepared by the academic curricula.

Moreover, among the educational initiatives described in the selected literature, the programs targeting adults primarily focus on the health care sector. Notwithstanding the relevance of nurturing emotional and social intelligence competencies in this specific organizational context, the studies analyzed do not consider the training initiatives implemented by many organizations in both the public and private sectors, aiming at providing executives with the ESCs to exercise their leadership successfully (Kark, 2011). Considering the huge amount of effort and resources invested by companies in various forms of training and development program to nurture emotional intelligence in their employees (Cherniss et al., 2010), scholars may further investigate how these initiatives vary in terms of objectives, content, methodologies, and behavioral change according to the different professional roles to whom the courses are addressed and the organizational contexts in which they are delivered. An interesting area of enquiry, that is also indicated by some scholars as one of the critique to the competency model, is represented by the analysis of how competency development programs should be tailored according to the specificity of the firm and job requirements as well as the industrial setting, since organizational context and group relationships assume a relevant role in influencing the learning process (Antonacopoulou and FitzGerald, 1996) and in supporting and encouraging individuals to undertake the learnt behaviors (Bolden and Gosling, 2006).

### Methodological Insights for Behavioral Competency Development

The review we conducted reveals that emotional and social competencies development should promote the adoption of a holistic approach to education that requires educators to rethink and innovate their own teaching methods and styles, shifting from a passive lecture approach to one that actively engages learners in activities designed for practice and experience. Indeed, in line with the experiential and whole person learning approach, a variety of teaching techniques have been proven to be effective in emotional and social education programs. These encompass self-reflection activities and meditation practice, but also interactive techniques such as coaching, role play, simulations, or the adoption of gamification.

Despite the contributions provided by these studies, further investigation is necessary to provide an in-depth analysis and systematization of the different learning techniques that can be adopted to develop ESCs. Future studies should identify and describe the conditions for their effective use and their association with specific competencies. For instance, if the techniques adopted during mindfulness-based interventions can be successfully proposed to enhance emotional self-awareness, self-control, and empathy, they may not be appropriate for other types of competency. Furthermore, even though the literature seems to converge on experiential learning theory (Kolb, 1984) as the pedagogical framework for designing competency development programs, studies do not address the issue of how each learning style adopted by individuals to practice the new behaviors calls for different techniques.

Another limitation we found in the analysis of the literature is related to the fact that many educational programs explicitly adopting the whole person learning approach limit the learning experience to just a set of competencies defined at institutional level. As suggested by other contributions, the effectiveness of a learning program is based on the intention of the individuals to undertake the development of specific competencies in line with their desired career goal (Cherniss and Adler, 2000; Boyatzis and Saatcioglu, 2008; Boyatzis and Cavanagh, 2018). Specifically, this different approach has been described in ICT, which advances self-directed learning by providing a theoretical framework for describing the essential components of desirable, sustainable change in one’s behavior, thoughts, feelings, and perceptions (Boyatzis, 2008). The facts that the change is desired and that it is sustainable represent two fundamental aspects of effective application of the ICT framework to emotional and social competencies. The change is desired by the person who wishes it, since learning goals are set intentionally by individuals to be coherent with their future personal and professional lives. The fact that, during the training experience, individuals intentionally identify the competencies they wish to learn represents an essential motivational driver to change their behavior successfully. Moreover, in contrast to the majority of training experiences, intentional change is also sustainable, in that it endures for a relatively long time. In line with this methodology, scholars should devote more attention to the development of didactic techniques which enable learners to become more aware of their overall portfolio of competencies at the beginning of the program and support them in identifying the ESCs needed to attain their desired future. Another feature of the analyzed training interventions is that the learning programs engage students with experiential techniques for promoting self-reflection and active learning in the classroom environment. Only a few initiatives provide the participants with a structured plan to practice the competencies in real life contexts in order to pursue a long-lasting change in their behavior. Adopting a more experimental approach to competency development offers individual more opportunity for self-discovery and the exploration of effective behavior in life and the organizational settings (Bolden and Gosling, 2006), and consequently it may favor a more sustainable change.

The intentional change approach seems to respond to the critique moved by Antonacopoulou and FitzGerald (1996) to competency development models. Indeed, the authors call for a more holistic, self-directed and experimental approach to competency development in line with the literature on adult education. Moreover, they pointed out that the competency learning initiatives mainly adopt a short-term logic in order to satisfy the current organizational needs. Instead, a future-oriented perspective is more in line with the concept of organization as dynamic and learning entities. In order to support organizations in their continuous improvement, educational institutions may play a pivotal role in identifying and developing those emotional and social intelligence competencies that will be central in the future jobs and are necessary to enable individuals to learn and adapt during periods of change.

Another limitation highlighted by the literature review is related to the types of learning objective proposed by these programs. As emerged from our analysis, social and emotional learning initiatives have primarily been designed for the development of a few types of emotional and social competency, specifically self-awareness, empathy, and some relationship management competencies such as teamwork, leadership, and conflict management. Apart some studies (Ballou et al., 1999; Boyatzis et al., 2002; Leonard, 2008; Rhee, 2008; Wheeler, 2008; Camuffo et al., 2009; Bonesso et al., 2015, 2018; Burch et al., 2016; Gilar-Corbí et al., 2018; Passarelli et al., 2018; Cronin et al., 2019; Dowling et al., 2019; Fernández-Pérez et al., 2019; Oi, 2019) that demonstrated a positive impact of the learning programs on a broad set of emotional, social and cognitive competencies, more efforts need to be devoted to the cognitive competencies and the self-management cluster, with the exception of the competencies of emotion regulation/self control frequently considered in the articles analyze. Future studies should advance the debate on the development of these skills that are assuming increasing relevance in the labor market, such as resilience, adaptability, problem-solving, and creative thinking, as highlighted by recent employer surveys (LinkedIn, 2019; QS Intelligence Unit, 2019). Finally, there is also a need for more robust studies which assess the effectiveness of ESC programs delivered in different cultural environments, since the majority of the studies have been conducted in the European and US contexts.

### Learning Outcomes and Assessment Methods

A limitation that almost all the studies analyzed have in common is that the outcomes in terms of individual behavioral change were measured immediately after the intervention had ended. Prior research suggests that the effects of many interventions diminish over time—the so-called honeymoon effect—and therefore future studies should devote more effort to investigating if the effects will endure post-intervention. Follow-up studies conducted several months after the end of the intervention could investigate the longer-term effects of the program on learners’ outcomes, as indicated in the SPR’s Standards Committee (Flay et al., 2005).

The review shows that even though the majority of the empirical works relies on the behavioral approach or third emotional intelligence stream in defining the competencies considered as learning outcomes, the same competency in different studies has been measured adopting diverse psychometric measures. This may limit the comparability of the learning experiences examined in the studies analyzed. This review was not intended to add to the debate on the different approaches to competency measurement, however, scholars should consider this issue as critical in the future research if they aim to compare how different learning methodologies affect the development of a specific competency. Scholars should motivate the decision to rely on a specific psychometric scale for measuring the behavioral competency that is the target of the training programs, reporting satisfying criteria for discriminant and convergent validity. Related to the issue of competency measurement, another critical issue that should be considered in designing competency development studies concerns the data collection technique. Indeed, self-report assessment in the pre- and post-intervention stages could be biased by the honeymoon effect or by a higher awareness in that specific competency matured during the learning program that allows the participant to assess the competency more objectively. As proposed by Boyatzis (2018) in the Stream 4 measures of emotional intelligence, the person’s behavior should be assessed by external raters who have the opportunity to observe the activation of the competencies in the work and life contexts pre and post the learning experience and during a long-term follow up session. Moreover, future research may measure the behaviors that the programs aim to develop relying not only on both self- and others’ evaluation through 360 assessments but also on direct observations, limiting the potential bias and the competing dynamics often ascribed to 360-degree appraisal tools used both for assessment and development purposes (Bolden and Gosling, 2006).

Only a few studies included in this review devote attention to other variables influenced by ESC development, focusing on entrepreneurial intention, individual wellbeing, career success, and satisfaction, and concentrating the attention primarily on students and educators. We suggest further consideration of other outcomes of the learning programs. For instance, an interesting line of enquiry is represented by the impact of the ESC learning programs on generating differences in individual performance in workplace settings, but also on employability and perceived employability, namely “the perceived ability to attain sustainable employment appropriate to one’s qualification level” (Rothwell et al., 2008: 2). Since employability depends on the optimal use of personal competences (Süß and Becker, 2013), and only anecdotal evidence has been provided on this relationship, an interesting area of enquiry is represented by investigating the effect of competency development on increasing individuals’ employability.

## Conclusion

Much has been written about the relevance of emotional and social competencies across professional roles and industrial settings, and our article systematically reviews the literature which has contributed so far to the debate on how behavioral competencies can be developed in the higher education and organizational environments. The contribution of our work is, on the one hand, to document the diversities and the commonalities among the theoretical and methodological frameworks adopted in several educational programs and, on the other hand, to identify major opportunities for their further advancement.

Education institutions and organizations should formally recognize the critical role of ESCs in contributing to individuals’ employability, job performance, and career success, investing appropriately in their development. This review represents the first attempt to systematize the methodologies of the educational programs for competency development and to assess their effectiveness in order to assist educators and executives in their ongoing efforts to equip students and employees with the relevant skills needed to achieve superior performance in the workplace. At the institutional level, policymakers should promote a dedicated agenda with concrete actions to equip people with emotional and social intelligence competencies—for instance, sponsoring and funding career pathway projects. Finally, the modern concept of the employment psychological contract, in which careers are conceived as more self-directed, value-driven, and boundaryless (Arthur et al., 2005; Gubler et al., 2014; Hall et al., 2018), spurs individuals to be proactive in undertaking lifelong learning through the identification of training opportunities which enable them to broaden their repertoire of emotional and social behaviors in line with their desired career path.

## Author Contributions

SB, FG, RZ, and RB contributed to the design of the study, organized and analyzed the database. SB, FG, RZ, and RB wrote the different sections of the manuscript. All authors contributed to manuscript revision, read and approved the submitted version.

## Conflict of Interest

The authors declare that the research was conducted in the absence of any commercial or financial relationships that could be construed as a potential conflict of interest.
